# Development of a Cost-Effective, Heme-Tolerant Bovine Muscle Cell for Cultivated Meat Production

**DOI:** 10.3390/foods14244348

**Published:** 2025-12-17

**Authors:** Yun Ok Oh, Chae Won Yu, Min Jeong Cha, Eun Ji Lee, Pil Kim, Suhwan Chang

**Affiliations:** 1 Department of Physiology, BK21 Project, Asan Medical Center, University of Ulsan College of Medicine, Seoul 05505, Republic of Korea; siyo565@gmail.com (Y.O.O.); hyloveej6@hanmail.net (E.J.L.); 2 Department of Biotechnology, The Catholic University of Korea, Bucheon 14662, Republic of Korea; kimp@catholic.ac.kr; 3 HemoLab Co., Ltd., Seoul 02174, Republic of Korea

**Keywords:** heme, muscle cell, adaptation, cultured meat, RNA-seq

## Abstract

One of the critical requirements for the production of artificial meat is to mimic the flavor of the original meat. Therefore, incorporation of heme has been proposed, but it is toxic when added at high concentrations in cell culture systems. Additionally, obtaining heme can be costly depending on the source. In this study, we aimed to support the growth of normal bovine muscle cells (BRMC-F2401, BRMCs) by introducing a bacterial extract obtained from a safe, high-heme-containing *Corynebacterium* species. The BRMCs exhibited heme toxicity when the bacterial heme level was >20 μM; however, they were adapted to stably proliferate with a 3 on–3 off culture scheme. RNA sequencing of the heme-adapted BRMCs showed gene expression changes, including upregulation of detoxification genes, *CYP1A1*, *CYP26B1*, and *SERPINB2*. The knockdown of these three genes increased heme sensitivity and reversed heme tolerance of the heme-adapted BRMCs. Additionally, ROS levels increased upon heme treatment, suggesting that ROS is an important factor in heme adaptation processes. Collectively, our study presents an affordable strategy to incorporate heme in cultured meat production and the mechanism underlying this process.

## 1. Introduction

In addition to the texture of the natural meat, flavor is one of the key factors for cultured meat to be accepted for consumption by the population [1]. Among various factors to reproduce original meat flavor, heme in blood is a unique and known essential factor [2]. However, there are limited ways to supply heme in a large quantity without using house stock and ensure cost effectiveness for incorporation in cultured meat. Studies to obtain heme from soy root nodules (Caco-2 cell model) [3], porcine blood (red blood cells) [4] and more recently, a plant-based heme containing protein (leghemoglobin) produced using yeast has been reported [5]. Additionally, we obtained heme from a safe bacterial species, *Corynebacterium*, using directional evolution [6]. *Corynebacterium* is a non-GMO, production host widely used, so there is no safety issue. Compared to other host such as yeast or plant for heme extract source, bacteria proliferate faster with low-cost media. Moreover, the *Corynebacterium* extract contains amino acids (nutrients) and is easy to obtain (mechanical disruption by sonication or other method) with simple process.

However, one of the major hurdles in heme incorporation is its toxicity caused by multiple processes. First, the excess free heme causes dysregulated lipid peroxidation [7], glutathione homeostasis [8], and mitochondrial ROS levels [9], and protein/DNA damage [10]. Second, molecular changes result in mitochondrial damage [9] and/or DNA fragmentation, which induce apoptosis or ferroptosis [11]. Finally, the cell death will drive organ-level pathogenicity in the lung, liver, kidney, cardiovascular system, and brain (CNS) [12]. To reduce heme toxicity, there are two major related pathways in vivo. One scavenges free heme using haptoglobin or hemopexin [13], and the other converts heme into biliverdin and free Fe using heme-oxygenase-1 [14]. However, if the heme level is above 10–50 μM in vitro or 10–20 μM in plasma [15], the excess amount of heme causes toxicity. To incorporate heme in the cultured meat for mimicking the natural flavor, therefore, it is therefore important to adapt cultured cells to a certain level of heme-containing media. We have successfully derived a normal, porcine cell line adapted to high heme-containing media. However, the cell line originates from the kidney, which limits its application. In this study, we aimed to develop a bovine muscle cell for future application in cultured meat production.

The bovine muscle cells are essential components for cultured beef production. Muscle stem cells (MuSC) are a common source for the cultured meat [16], but they have limited proliferation potential [17]. To overcome this, an immortalized muscle stem cell has been developed by introducing hTERT and CDK4 [18]. According to patent information, several bovine cells (B10M-t3 and B4M-t6S1) are adopted in suspension culture under serum-free conditions. A recent publication showed that the addition of extracellular heme protein, such as myoglobin, increases the growth of bovine muscle stem cells [19], confirming that heme scavenging influences the proliferation of muscle cells. The addition of heme mimics the original flavor of natural meat [20]. Therefore, the adaptation of bovine muscle cells to heme is a preferred condition for the final cultured meat produced in serum-free media. In this study, we used a stem cell-derived, normal bovine muscle cell line and adapted it in media containing heme obtained from *Corynebacterium*.

## 2. Materials and Methods

### 2.1. Cell Culture and Media

Bovine fetal muscle mesenchymal stem cells (BRMC-F2401) were provided by MK-biotech Inc. (Daejeon, Republic of Korea) and maintained in skeletal muscle cell growth medium (MK-SFM, MK-biotech) supplemented with 10% fetal bovine serum (FBS; Hyclone, Logan, UT, USA) and 1% penicillin/streptomycin (P/S; Hyclone). Cells were proliferated for more than three passages in MK-SFM, after which the medium was gradually replaced with an increasing proportion of complete DMEM. HEK293 cells were cultured in DMEM supplemented with 10% FBS and 1% P/S. All cells were maintained in a humidified incubator at 37 °C with 5% CO_2_. Medium was refreshed every 2–3 days, and cells were sub-cultured with 0.25% trypsin–EDTA prior to reaching 85–90% confluence.

### 2.2. Preparation and Quantification of the Heme Extract

To prepare the heme extract, 10 g of dried heme-overexpressing *Corynebacterium* was suspended in 40 mL of distilled water in a 50 mL conical tube. The suspension was sonicated using a pulse sonicator (VCX130, Sonics & Materials Inc., Newtown, CT, USA) at 100% amplitude for 30 min with 2 s on/5 s off cycles to minimize temperature increase. The lysate was centrifuged at 4000 rpm for 20 min at 4 °C, and the supernatant was transferred to 1.5 mL microtubes and centrifuged again at 14,000 rpm for 15 min at 4 °C. The clarified supernatant was collected in a glass bottle, and glycerol was added to a final concentration of 5%. Following autoclaving, the solution was dispensed into 1 mL aliquots and stored at 4 °C until use.

The concentration of the heme extract was measured using a heme assay kit (MAK316, Sigma-Aldrich, St. Louis, MO, USA) following the manufacturer’s protocol. Total protein concentration was also determined using a BCA assay (Thermo Fisher Scientific, Waltham, MA, USA) and expressed relative to the corresponding unit of heme.

### 2.3. Cell Adaptation to High-Heme Conditions

BRMC-F2401 cells (BRMCs) were initially cultured in DMEM supplemented with 10% fetal bovine serum (FBS). To establish serum-reduced conditions, the serum concentration was progressively lowered to 5%, 2.5%, and finally 1% FBS. Thereafter, all adaptation experiments were performed in DMEM containing 1% FBS. For heme adaptation, cells were exposed to stepwise increasing concentrations of heme (10, 20, and 40 μM). Each exposure period lasted 72 h (“on” phase), followed by a 72 h recovery period (“off” phase) in heme-free DMEM containing 1% FBS. This “3 on–3 off” adaptation cycle (72 h exposure + 72 h recovery) was repeated three consecutive times to promote stable cellular adaptation. After completion of the third cycle, cells were maintained in DMEM containing 1% FBS with the designated heme concentration for an additional 72 h prior to functional assays. Control cells were cultured in parallel under identical serum-reduced conditions without heme exposure.

### 2.4. Cell Growth and Viability Assay

The effects of serum concentration and heme cytotoxicity on cell proliferation were assessed using a WST-8 assay kit (Quantimax, Biomax, Gyeonggi-do, Republic of Korea). To examine the effect of FBS concentration, BRMCs were seeded in 96-well plates at a density of 5 × 10^3^ cells per well. Cells were cultured in MK-SFM, DMEM, or RPMI media containing decreasing concentrations of FBS, incubated at 37 °C for 72 h, and cell proliferation was subsequently measured using the WST-8 assay according to the manufacturer’s instructions. To assess cell viability under heme exposure, BRMCs were seeded in 96-well plates at a density of 1 × 10^4^ cells/well and incubated in DMEM supplemented with 1% FBS at 37 °C for 24, 48, or 72 h. At the indicated time points, WST-8 reagent was added to each well at 1/10 of the culture volume, followed by incubation for 2 h at 37 °C. After gentle mixing, absorbance was measured at 450 nm using a microplate reader (Synergy HT Multimode Microplate Reader, BioTek, Winooski, VT, USA). Relative cell viability (%) was determined by normalizing the absorbance of the treated cells and the control cells to that of the blank wells using the following formula:

% Cell Viability = [(A_treatment_ − A_blank_)/(A_control_ − A_blank_)] × 100%

### 2.5. Construction and Cloning of pLKO.1-TRC shRNA Vectors

BRMCs were stably transfected with a pLKO.1-TRC cloning vector (plasmid #10878; Addgene, Watertown, MA, USA) carrying gene-specific shRNA sequences. Target-specific shRNA sequences were designed according to The RNAi Consortium (TRC) guidelines, and corresponding oligonucleotide sequences are listed in Table S1. The pLKO.1-TRC vector was digested with AgeI and EcoRI restriction enzymes and purified using a gel extraction kit. For each shRNA targeting *CYP1A1*, *CYP26B1*, and *SERPINB2*, complementary oligonucleotides with appropriate overhangs were synthesized, annealed, and ligated into the AgeI and EcoRI sites of the pLKO.1 vector. The ligation mixtures were transformed into competent *E. coli*, and positive clones were screened and verified using PCR amplification.

### 2.6. Lentiviral-Mediated shRNA Knockdown

Recombinant lentiviruses were generated by co-transfecting HEK293T cells with the pLKO.1-shRNA plasmid and lentiviral packaging vectors, psPAX2 and pMD2.G, using Lipofectamine 3000 (L3000075; Invitrogen, Carlsbad, CA, USA), following the manufacturer’s instructions. The transfection medium was replaced with fresh complete medium 6 h post-transfection. Lentiviral supernatants were collected at 24 and 48 h, filtered through a 0.45-μm syringe filter to remove cellular debris, and immediately used for transduction. BRMCs were infected with the recombinant lentivirus in the presence of 5 μg/mL of hexadimethrine bromide (H9268; Thermo Fisher Scientific) for 2 days. After a 3-day recovery period, transduced cells were selected with 2 μg/mL of puromycin for 3–5 days. Knockdown efficiency was subsequently assessed using RT-PCR and Western blotting.

### 2.7. RNA Isolation and RT–PCR

Total RNA was isolated using Tri-RNA reagent (Invitrogen) according to the manufacturer’s protocol. First-strand cDNA was synthesized using the PrimeScript RT reagent kit (TaKaRa, San Jose, CA, USA). Quantitative RT-PCR was performed with SYBR Green (AMPIGENE qPCR Green Mix Lo-ROX, Enzo Life Sciences, Farmingdale, NY, USA) on a CFX Connect Optics Module (Bio-Rad, Hercules, CA, USA). Primers used in this study are listed in Table S1. GAPDH served as the reference gene for normalization, and relative gene expression levels were calculated using the 2^−ΔΔCT^ method.

### 2.8. Western Blotting

Cells were lysed in RIPA buffer (Biosesang, Gyeonggi-do, Republic of Korea), and protein concentrations were determined using the BCA assay. Equal amounts of protein (12 μg) were separated using 12% SDS–PAGE and transferred to nitrocellulose membranes (Thermo Fisher Scientific). Membranes were blocked with 5% skim milk in TBST for 1 h at room temperature and incubated overnight at 4 °C with the following primary antibodies: CYP1A1 (orb578044, Biorbyt, Cambridge, UK), CYP26B1 (NBP1-33476, Novus Biologicals, Centennial, CO, USA), SERPINB2 (orb584271, Biorbyt), THBS1 (sc-59887, Santa Cruz Biotechnology, Dallas, TX, USA), GST (K200006M, Solarbio, Beijing, China), and β-actin (sc-47778, Santa Cruz Biotechnology). After washing, membranes were incubated with HRP-conjugated secondary antibodies for 2 h at room temperature. Protein bands were visualized using an ECL detection kit (Life Technologies, Seoul, Republic of Korea) and imaged with a Davinch-Chemi Imager (CAS-400SM, Core Bio, Seoul, Republic of Korea).

### 2.9. Determination of Intracellular ROS Generation

Intracellular ROS generation was evaluated fluorometrically to assess the detoxification capacity of heme-adapted BRMCs against heme-induced oxidative stress.

Heme-adapted BRMC-F2401 (F1-H20) cells and shRNA knockdown (KD) cells targeting *CYP1A1*, *CYP26B1*, and *SERPINB2* were seeded in white 96-well plates at a density of 1 × 10^4^ cells/well in DMEM supplemented with 1% FBS and incubated at 37 °C for 24 h. Cells were then exposed to 40 μM heme extract for an additional 24 h. After treatment, cells were incubated with 20 μM 2′,7′-dichlorodihydrofluorescein diacetate in DPBS for 30 min at 37 °C in the dark. Following incubation, cells were washed with phosphate-buffered saline (PBS) to remove excess dye, and fluorescence intensity was measured at excitation and emission wavelengths of 490 and 525 nm, respectively, using a Synergy HT multimode microplate reader (BioTek).

For fluorescence imaging, F1-H20 and shRNA KD cells were seeded in 12-well plates at a density of 1 × 10^5^ cells/well in DMEM containing 1% FBS and incubated at 37 °C for 24 h. Cells were then treated with or without 40 μM heme extract, and DCF fluorescence (DCF-FL) images were acquired using a fluorescence microscope.

### 2.10. Statistical Analysis

All experiments were performed at least three times independently. Data are presented as means ± SD. Statistical analyses were performed using one-way ANOVA in GraphPad Prism 8.0 (GraphPad Software, Boston, MA, USA). Differences were considered statistically significant at *p* < 0.05.

## 3. Results

### 3.1. Optimization of Culture Condition for the BRMC-F2401 Bovine Muscle Cell Line

**Figure 1:**
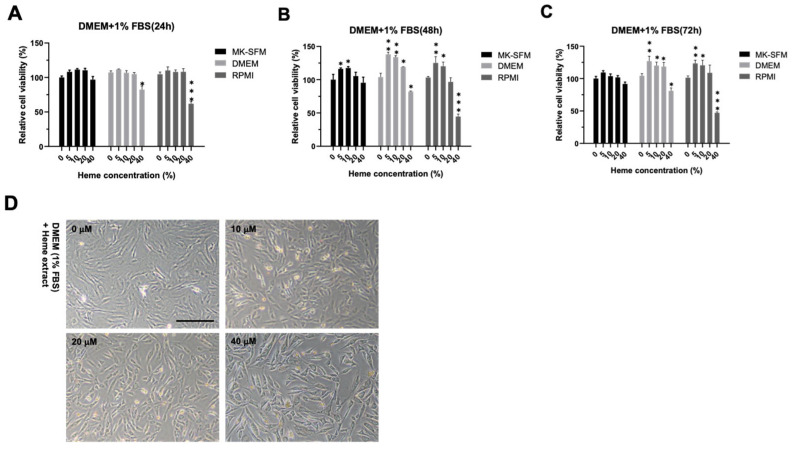
Heme-containing bacterial extract with BRMC-F2401 cells (BRMC) causes reduced proliferation with morphological changes. (**A**–**C**) Time-course, relative cell viability of BRMCs cultured in DMEM or RPMI supplemented with 1% FBS plus varying concentrations of heme obtained from *Corynebacterium* extract. Values are expressed as mean ± SD. (**D**) Representative images of BMRCs cultured with varying doses (0–40 µM) of heme-containing DMEM. * *p* < 0.05, ** *p* < 0.01, *** *p* < 0.001. Scale bar: 200 mm. FBS, fetal bovine serum.

The cost of serum-free, conditioned media for BRMCs was inadequate for large-scale production; therefore, we tested whether the BRMCs can be cultured under general media with reduced serum conditions. MK-SFM supported the growth of BRMCs without serum, but 2.5% serum showed better BRMC growth (Figure S1a). We found a significant reduction in the proliferation of BRMC when cultured in DMEM or RPMI with reduced serum (Figure S1b,c). However, when heme extract from the *Corynebacterium* was added in the 1% reduced serum condition, we observed that BRMCs endured up to 20 µM of heme for 72 h (Figure 1A–C), with no difference compared with the base medium. In 40 µM of heme, cell death was observed (Figure 1A–C) with a slightly enlarged cell (Figure 1D). Additionally, we observed that BRMCs become stretched out in the DMEM or RPMI with high serum concentrations (5–10%), but this change was not observed with 0% FBS (Figure S1d). The transient treatment of heme caused cell death; therefore, we decided to adapt BRMCs with heme under DMEM or RPMI with 1% serum.

### 3.2. Derivation of Heme-Adapted BRMCs by Continuous Culture

**Figure 2:**
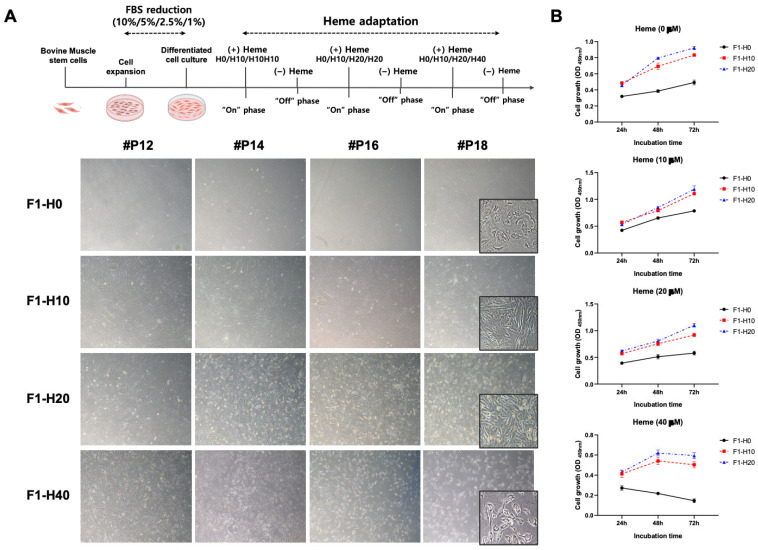
Adaptation of BRMCs under the high-heme condition using 3 on–3 off methods and its validation for heme tolerance. (**A**) Culture scheme (upper part) with representative images of BRMCs under the heme adaptation process (lower part). The cells were continuously cultured in 0–40 µM of heme-containing bacterial extract up to 18 passages. At P18, the inset images on the corner of each image show morphological differences as well as cell density. (**B**) Time course proliferation of heme-adapted BRMCs (BRMC-ha, denoted as F1H10 (in red) and F1H20 (in blue)) under 0–40 µM of heme, compared with the control (in black). BRMC, BRMC-F2401 cell.

We used a 3 on-3 off method to establish heme-adapted BRMCs under 20 and 40 µM of heme (denoted as BRMC-ha). As shown in Figure 2A, the addition of heme in the 1% serum condition (up to 20 µM) supported BRMC growth. The BRMCs cultured with 1% FBS without heme (F1H0) showed reduced growth with senescence-like morphology (Figure 2A). Additionally, reduced growth and cell death of BRMCs were observed on P18 with 40 mM of heme; however, stable growth was observed at 20 µM of heme (BRMC-ha). To confirm the adaptation at the high heme concentration, we measured the growth of BRMC-ha cells under 10–40 µM of heme. Figure 2B shows that two BRMCs that adapted under either 10 or 20 µM of heme (F1H10 or F1H20, respectively) exhibited stable and superior growth compared with that of the control under 10–20 µM heme-containing media.

### 3.3. RNA Sequencing (RNA-Seq) Reveals Differentially Expressed Genes in Heme-Adapted BRMC-Ha Cells

**Figure 3:**
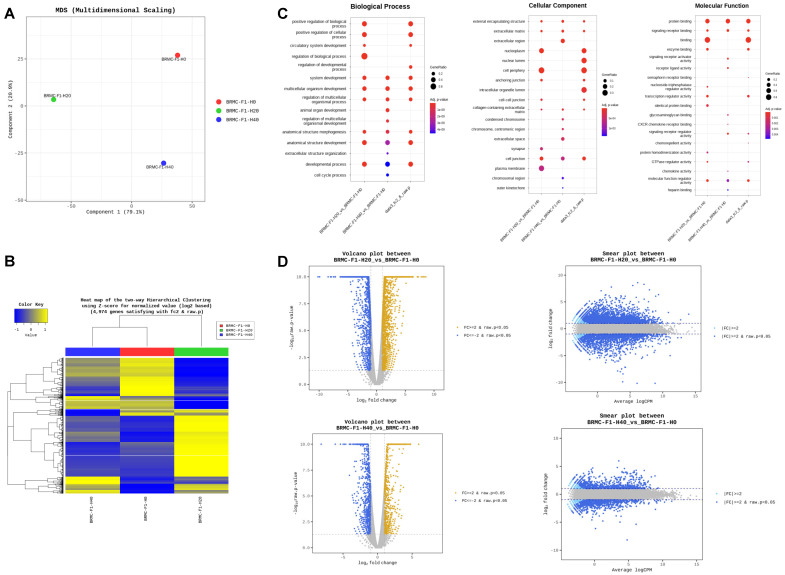
Analysis of differentially expressed genes (DEGs) based on RNA sequencing in heme-adapted BRMCs. (**A**) MDS plot comparing control (F1-H0) and heme-adapted BRMCs (F1-H20/F1-H40). (**B**) The heatmap of the two-way hierarchical clustering analysis in F1-H0 and F1-H20/F-H40. (**C**) Biological process, cellular component, and molecular function terms of KEGG and GO analysis of DEGs. (**D**) Volcano (on left) and smear (on right) plots presenting DEGs with *p*-values. BRMC, BRMC-F2401 cell.

To understand the molecular mechanism of the adaptation for high-heme-containing media, we performed RNA-seq for the heme-adapted BMRC cells (on P12). Figure 3A shows PCA of the control (BMRC-F1 H0), and BRMC-ha cell under 20 mM (BMRC-F1-H20) and 40 mM of heme (BMRC-F1-H40) cultured with 1% of FBS, indicating clear separation of the three samples in their expression profile. Subsequent analysis of comparative expression analysis revealed different expression signatures among the three groups, presenting BMRC-F1-H20 as a distinct group (Figure 3B, Table 1 for the top gene list) compared with the control group (F1H0). Some of the genes were related to heme binding, export, and biogenesis processes (Table 2). The pathway analysis among the three groups identified several biological processes, cellular components, and molecular functions among the heme-adapted BMRCs (Figure 3C, Table S2). Volcano or smear plot analysis for the three datasets also confirmed the expression changes triggered by high heme adaptation (Figure 3D). Additional analysis demonstrated increased expression of genes associated with cellular heme handling and redox modulation, including HMOX1 (HO-1), NQO1, NQO2, FECH, FTN1, BACH1, GPX1 and GSTT1 [10,11,12,13], following heme adaptation (F1-H20 versus F1-H0) (Supplementary Table S3). Other data from the RNA-seq analysis are provided in Figure S2. Collectively, these results demonstrate that the heme adaptation of BMRCs is accompanied by distinct gene expression changes.

### 3.4. Confirmation of the Gene Expression Changes in the BRMC-Ha Based on Heme Detoxification

**Figure 4:**
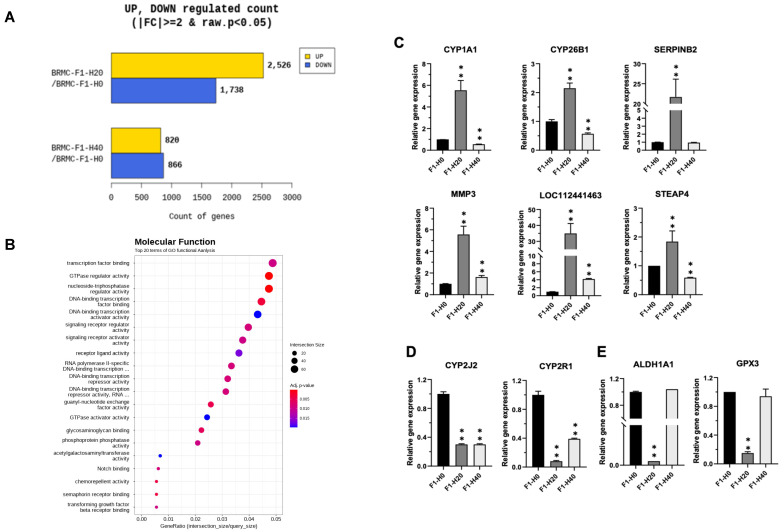
Distinct gene expression signature in heme-adapted BRMCs. (**A**) Analysis of biological functions associated with differentially expressed genes between control (F1-H0) and heme-adapted (F1-H20/F-H40) cells. (**B**) Gene ontology analysis highlighting altered key molecular functions of the BMRC-ha cells. (**C**–**E**) RT-PCR validation of upregulated (**C**), downregulated (**D**), and dynamically expressed genes (**E**) in heme-adapted cells (F1-H20/F-H40). Values are expressed as mean ± SD; ** *p* < 0.01 as compared to control (F1-H0). BRMC, BRMC-F2401 cell.

Among a set of differentially expressed genes (Figure 4A), we further validated expression changes for specific genes, possibly implicated in heme detoxification. The BMRC-F1-H20 cell showed more expression changes than those of BMRC-F1-H40 cells. Differentially expressed gene analysis revealed the enriched transcription factor or signaling receptor activity of BMRC-F1-H20 (Figure 4B), suggesting a signaling pathway triggered by heme adaptation involves transcriptional activation. All other results for the pathway or gene-ontology analysis are provided in Figure S3. Genes involved in one of the major detoxification systems of the mitochondrial P450 complex, *CYP1A1* and *CYP26B1*, were upregulated in the BMRC-F1-H20 cells (Figure 4C). Notably, STRING analysis showed the interaction of these two genes (Figure S4), suggesting functional cooperation in the heme detoxification process. Moreover, we observed upregulation of *SERPINB2, MMP3*, and *STEAP4*, and downregulation of *CYP2J2*, *CYP2R1*, *ALDH1A1*, and *GPX3* in BMRC-F1-H20 cells (Figure 4D,E). Some of the gene expression changes in BRMC-F1-H20 were not evident or unchanged. These data indicate that the stable adaptation of BMRCs to high heme media causes specific gene expression changes involved in the detoxification system.

### 3.5. Upregulated CYP1A1, CYP26B1, and SERPINB2 Are Required for the Adaptation of Heme Toxicity in the BRMC-Ha Cells

**Figure 5:**
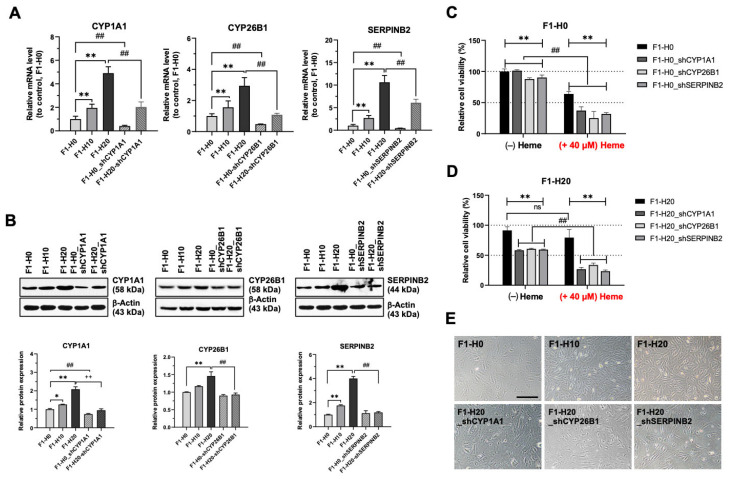
shRNA knockdown of the upregulated genes in BRMC-ha cells sensitizes the BRMCs to heme treatment. (**A**,**B**) Efficacy of shRNA-mediated knockdown of the three upregulated genes in BRMC-ha cells, assessed using (**A**) RT-PCR and (**B**) Western blotting. (**C**) The growth of two BRMC-ha cells was measured under increasing heme concentrations. (**D**,**E**) Comparison of cell viability (**D**) and representative images (**E**) was evaluated under 40 µM heme at 72 h. Values are expressed as mean ± SD; ns: not significant, ^##^ *p* < 0.01 (comparison between Heme(+/−) groups; ** *p* < 0.01 as compared with the control, scale bar: 200 mm. BRMC, BRMC-F2401 cell; BRMC-ha, heme-adapted BRMC.

To address the functional implications of the gene expression changes, we introduced shRNA knockdown of specific genes upregulated in BMRC-ha cells. Figure 5A shows the RNA level of *CYP1A1*, *CYP26B1,* and *SERPINB2,* which are upregulated in Figure 4C. Compared with the control cells (F1H0), the heme-adapted cells (F1H10 and F1H20) showed increased expression of the three genes (Figure 5A). In these cells, we introduced shRNA for the three genes (*CYP1A1*, *CYP26B1*, and *SERPINB2*) that restored the upregulated gene level to the control (Figure 5A). The RNA level changes were also confirmed in the protein, as shown in Figure 5B. Using these cells, we examined whether the three upregulated genes contributed to cell viability or proliferation during the heme adaptation process. Figure 5C,D shows that the knockdown of *CYPA1*, *CYP26B1*, or *SERPINB2* sensitized the control or BMRC-ha cells to the heme treatment, indicating the upregulation of the three genes is required for overcoming the toxicity caused by heme treatment. A dose-dependent proliferation analysis for the same set of cells confirmed the results (Figure S5). Moreover, Figure 5E supports this conclusion by showing that the cells with stable knockdown of the three upregulated genes proliferated less with morphological changes (Figure 5E).

### 3.6. BMRC-Ha Cells Attenuate Heme-Mediated ROS Generation via the Upregulation of Genes Involved in the Detoxification Process

**Figure 6:**
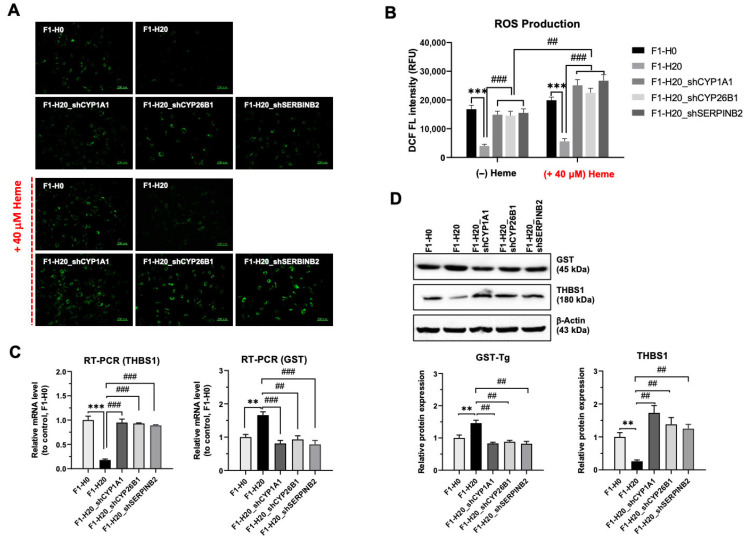
BMRC-ha cells attenuate heme-mediated ROS generation via the upregulation of genes involved in the detoxification process. (**A**) Representative images of ROS signal (green) in the control (F1H0), BRMC-ha (F1H20), and BRMC-ha with knockdown of the three detoxification genes under control media (upper panels) or heme-treated cells (lower panels). (**B**) Graph showing the quantitative analysis of intracellular ROS levels in Panel (**A**). (**C**) RT-PCR and (**D**) Western blot analyses for heme-binding, ROS modulator, THBS1, or GST. The lower graphs on (**D**) present quantitation results of the western signal. Values are expressed as Mean ± SD; ^##^ *p* < 0.01, ^###^ *p* < 0.001 (comparison for knock-down cells); ** *p* < 0.01, *** *p* < 0.001 as compared to control. scale bar: 200 mm. BRMC, BRMC-F2401 cell; BRMC-ha, heme-adapted BRMC.

One of the mechanisms of heme toxicity involves upregulated ROS [7]. When the BRMC-ha cells were labeled with a ROS probe, they showed decreased intracellular ROS levels (Figure 6A). Importantly, the cells with the knockdown of three detoxification genes in Figure 5 showed restored ROS levels (Figure 6A,B). In the heme-adapted cells, the high level of ROS could be removed by antioxidant genes, including *GST* and *THBS1* [21]. We examined whether the BRMC-ha cells express altered GST or THBS1, known to regulate ROS. In Figure 6C, the RNA level of GST was upregulated while *THBS1* was downregulated in F1-H20 BRMC-ha cells. Notably, the stable knockdown of the three genes restored the upregulated GST or downregulated *THBS1* level, suggesting that the detoxification includes reducing the ROS level and thereby ROS-induced damage. To confirm the altered RNA expression of the GST and *THBS1* at the protein level, we performed Western blotting. Figure 6D shows the upregulated GST and downregulated *THBS1* in BRMC-F1-H20 cells that were restored by the knockdown of the three detoxification genes.

## 4. Discussion

*CYP1A1* and *CYP26B1* are components of the cytochrome P450 complex, which is involved in various detoxification processes [22]. Notably, heme is a prosthetic group of the *CYP1A1*, but there is no evidence supporting the direct detoxification of heme by *CYP1A1*. In our study, the knockdown of *CYP1A1* reduced the proliferation of BMRCs under heme-containing media. However, *CYP26B1* plays a role in retinoic acid metabolism [23] but not in heme detoxification. *THBS1* knockdown inhibits inflammatory damage and oxidative responses in a human bronchial epithelial cell line [21]. Additionally, our previous study showed it is downregulated in heme-adapted porcine cells, consistent with BRMC results in Figure 5C. The elevation of HMOX1 reflects enhanced activity of heme-sensing transcriptional circuits, whereas increased FTN1 expression is compatible with a greater cellular propensity for iron buffering [24,25]. In parallel, the induction of NQO1 and GPX1 suggests a shift toward strengthened intracellular redox control mechanisms [26]. The increased abundance of BACH1, a transcriptional regulator linked to the heme–redox interface, points to reorganization of heme-responsive gene networks rather than uniform activation of antioxidant signaling [27]. Although CYP1A1, CYP26B1, and SERPINB2 were also responsive at the transcript level, their regulation is considered reflective of broader stress-associated transcriptional plasticity and is not interpreted as evidence of a direct, causal role in heme tolerance [28]. In contrast, in cells adapted to higher heme levels (F1-H40), the expression of HMOX1-related genes remained elevated relative to F1-H0 but was reduced compared with F1-H20. This attenuated response likely reflects a diminished induction of redox defense mechanisms due to heme-induced cytotoxic stress, suggesting that excessive heme exposure imposes limits on adaptive transcriptional remodeling [29]. Together, these transcriptomic patterns indicate that heme exposure is accompanied by coordinated transcriptional adjustments across pathways tied to Ho-1-associated heme catabolism, ferritin-mediated iron buffering, and Nrf2-linked antioxidant defenses, supporting adaptive cellular redox regulation under moderate heme-enriched conditions.

We observed F1H0 cells undergo senescence-like morphology, typically observed in primary cells under serum starvation [30]. Notably, the addition of heme-containing *Corynebacterium* extract prevented this phenotype, resulting in fibroblast-like growth (Figure 2A). Considering the bacterial extract contains nutrients, including amino acids, sugar, and lipids [31], it can be used in low serum conditions. Therefore, in addition to providing heme, the role of the bacterial extract as a nutrient in low or serum-free conditions should be further studied.

In the RNA-seq analysis of heme-adapted BRMCs, we observed unexpected differences between F1H20 and F1H40 cells (Figure 3A). The F1H40 cells were unable to be maintained after P18 (Figure 2A); therefore, we think the detoxification process was not enough for F1H40 cells. RT-PCR analysis of detoxification genes upregulated in F1H20 were not changed in F1H40 on P12 (Figure 4C). Similarly, some of the downregulated genes in F1H20 were unchanged or marginally downregulated (Figure 4D,E). It is unclear whether the exposure to the high heme concentration is the cause of this difference, but it may accelerate cell death or senescence that can affect gene expression patterns represented by the remaining cells. Additionally, the bacterial extracts can trigger gene expression changes independently of heme. Indeed, our previous study with porcine kidney cells showed that the bacterial heme extract induced a set of immune response genes. In this study, we did not find such a signature (Figure 3C and Supplementary Figure S3A), but there can be other pathways activated by the bacterial extract. It is also possible that the non-heme component of the bacterial extract can cause the expression difference between F1H20 and F1H40 cells. Further study using *Corynebacterium* with a low level of heme will clarify this point.

Current cultivated meat systems seek to regulate the proliferation and differentiation of muscle stem cells into myofibers under animal–component–free conditions [32]. The myogenic regulatory factors (MRFs) Myf5, MyoD, and MyoG are widely recognized as central regulators of myogenic lineage progression and muscle maturation [33,34]. In cultivated meat applications, these factors are commonly used as molecular indicators of myogenic differentiation and skeletal muscle formation [35]. To examine whether heme-adaptation was accompanied by alterations in myogenic differentiation, we analyzed the expression of Myf5, MyoD, and MyoG in heme-adapted cells using RT–PCR (Supplementary Figure S6). The result shows that the adaptation for low to intermediate concentrations of heme extract (F1-H10 and F1-H20) did not produce detectable changes in the expression of myogenic markers compared with untreated controls (F1-H0). In contrast, cells exposed to a higher concentration of heme proteins (F1-H40) displayed reduced expression of myogenic markers relative to the control group. This observation is consistent with the finding that excessive heme levels exert inhibitory effects on myogenic processes, potentially related to oxidative stress associated with iron reactivity [36].

## 5. Conclusions

This study provides a new insight into the cost-effective production of cultured meat, using a safe *Corynebacterium* extract containing high heme. Further studies are required to adapt the BRMCs under serum-free conditions in suspension or 3D culture with affordable methods. These efforts will complement this study to effectively produce cultured meat with a more natural flavor.

## Figures and Tables

**Table 1:** List of selected genes differentially expressed related to detoxification of heme toxicity in heme-adapted BRMC-F2401 cells.

No.	Gene_ID	Transcript_ID	Gene_Symbol	Description	BRMC-F1-H20/	BRMC-F1-H20/	BRMC-F1-H20/	BRMC-F1-H20/
					BRMC-F1-H0.fc	BRMC-F1-H0.logCPM	BRMC-F1-H0.raw.pval	BRMC-F1-H0.bh.pval
1	505184	NM_001192051	SERPINB2	serpin family B member 2	310.384251	7.122725	2.14E−163	1.19E−159
2	281309	NM_001206637	MMP3	matrix metallopeptidase 3	382.803057	6.166615	7.78E−152	3.24E−148
				(stromelysin 1, progelatinase)				
3	112441463	XM_024975704	LOC112441463	interstitial collagenase-like	66.893402	8.918018	4.36E−123	9.08E−120
4	281615	NM_174239	ALDH1A1	aldehyde dehydrogenase 1 family member A1	−46.095674	6.782363	5.11E−104	6.08E−101
5	281210	NM_174077,	GPX3	glutathione peroxidase 3	−11.536539	3.511371	1.17E−44	1.30E−42
		NR_138142						
**No.**	**Gene_ID**	**Transcript_ID**	**Gene_Symbol**	**Term_name**	**BRMC-F1-H20/**	**BRMC-F1-H20/**	**BRMC-F1-H20/**	**BRMC-F1-H20/**
					**BRMC-F1-H0.fc**	**BRMC-F1-H0.logCPM**	**BRMC-F1-H0.raw.pval**	**BRMC-F1-H0.bh.pval**
6	538861	XM_002686859	STEAP4	heme binding	54.313319	2.768096	3.27E−63	9.54E−61
7	282870	XM_002696635,	CYP1A1	heme binding	15.99953	3.652174	3.27E−52	5.39E−50
		XM_005222018						
8	510406	XM_002707809	CYP2J2	heme binding	−12.126655	2.071745	5.32E−35	3.64E−33
9	541302	NM_001076267,	CYP2R1	heme binding	−4.60079	3.860629	4.36E−22	1.27E−20
		XM_005216056,						
		XM_005216057,						
		XM_005216059,						
		XM_010812511,						
		XM_024975514						
10	282211	NM_174529,	CYP2D14	heme binding	−2.224938	3.59268	1.95E−07	1.28E−06
		XM_010805743						

**Table 2:** List of top differentially expressed genes related to heme binding in heme-adapted BRMC-F2401 cells.

Source	term_id	term_name	adjusted_*p*_value	intersection_size	Gene_ID	Transcript_ID	Gene_Symbol	BRMC-F1-H20/	BRMC-F1-H20/	BRMC-F1-H20/	BRMC-F1-H20/	N_BRMC-F1-H0	N_BRMC-F1-H20
								BRMC-F1-H0.fc	BRMC-F1-H0.logCPM	BRMC-F1-H0.raw.pval	BRMC-F1-H0.bh.pval		
GO:MF	GO:0020037	heme binding	0.76595423	11	538861	XM_002686859	**STEAP4**	54.313319	2.768096	3.27E−63	9.54E−61	0.312384	3.831677
GO:MF	GO:0020037	heme binding	0.76595423	11	282870	XM_002696635,	**CYP1A1**	15.99953	3.652174	3.27E−52	5.39E−50	1.304012	4.616819
						XM_005222018							
GO:MF	GO:0020037	heme binding	0.76595423	11	282022	NM_001105323, XM_024998320, XM_024998321, XM_024998322	**PTGS1**	11.120892	5.580917	2.16E−50	3.21E−48	3.151377	6.471008
GO:MF	GO:0020037	heme binding	0.76595423	11	510406	XM_002707809	**CYP2J2**	−12.126655	2.071745	5.32E−35	3.64E−33	3.133145	0.71297
GO:MF	GO:0020037	heme binding	0.76595423	11	282021	NM_174444, XM_015474114, XM_015474115	**PTGIS**	−5.568505	4.904741	1.43E−28	6.84E−27	5.69454	3.33898
GO:MF	GO:0020037	heme binding	0.76595423	11	541302	NM_001076267,	**CYP2R1**	−4.60079	3.860629	4.36E−22	1.27E−20	4.635735	2.628638
						XM_005216056,							
						XM_005216057,							
						XM_005216059,							
						XM_010812511,							
						XM_024975514							
GO:MF	GO:0020037	heme binding	0.76595423	11	282023	NM_174445	**PTGS2**	3.943702	5.65262	4.19E−20	1.07E−18	4.41456	6.342817
GO:MF	GO:0020037	heme binding	0.76595423	11	540573	NM_001192745	**STC2**	−5.074452	1.717307	1.05E−17	2.20E−16	2.69734	1.056233
GO:BP	GO:0015886	heme transport	0.27742268	2	511097	NM_001079585	**SLC46A1**	4.211165	1.922604	1.03E−14	1.63E−13	1.282115	2.814889
GO:BP	GO:0006784	heme A biosynthetic process	0.23434953	3	534286	NM_001101154, XM_024982845, XM_024982846	**ALAS1**	−2.442743	6.471196	7.66E−10	7.02E−09	6.987339	5.715116
GO:BP	GO:0006784	heme A biosynthetic process	0.23434953	3	517811	NM_001076861, XM_024985622, XM_024985623	**COX15**	2.25615	4.449531	5.34E−08	3.83E−07	3.846963	4.964013
GO:BP	GO:0006784	heme A biosynthetic process	0.23434953	3	281158	NM_174054	**FECH**	2.297128	3.517908	8.28E−08	5.75E−07	2.985136	4.078452
GO:MF	GO:0020037	heme binding	0.76595423	11	282211	NM_174529,	**CYP2D14**	−2.224938	3.59268	1.95E−07	1.28E−06	4.139594	3.082574
						XM_010805743							
GO:BP	GO:0097037	heme export	0.26278276	1	533317	NM_001206019, XM_010813570, XR_003029610	**FLVCR1**	2.074857	4.163325	1.20E−06	6.93E−06	3.657981	4.650661
GO:MF	GO:0020037	heme binding	0.76595423	11	504769	NM_001075173,	**CYP2B6**	−2.778364	−0.648631	0.00036633	0.00129398	0.934511	0.407831
						XM_005218914							

## Data Availability

The original contributions presented in the study are included in the article/Supplementary Material; further inquiries can be directed to the corresponding author.

## Supplementary Materials

The following supporting information can be downloaded at https://www.mdpi.com/article/10.3390/foods14244348/s1, Figure S1. (A) The growth of BRMC in MK-SFM media for 2 and 4 days under various serum conditions. (B) Comparison of BRMC bovine muscle cell proliferation under different culture medium supplemented with increasing amount of fetal bovine serum (FB S). (C) The proliferation of BRMC in three different growth media with decreasing FBS concentration. (D) Morphological changes of cultured BRMC-F2401 cells in three different growth media with serum-free, 5% and 10% FBS concentration. Figure S2. Quality control and analysis of RNA-Seq data from control and heme-adapted BRMC-F2401 cells. (A) Distribution of TMM-normalized values for the pair F1-H0 vs. F1-H20. (B) Boxplots showing expression distributions across samples based on percentiles of raw counts, log_2_;-transformed read counts, and TMM-normalized values in F1-H0, F1-H20, and F1-H40. (C) Correlation matrix illustrating expression similarity among all samples (F1-H0, F1-H20, and F1-H40). (D) Dendrogram generated from hierarchical clustering of QC-filtered expression data, showing sample similarity between F1-H0 and F1-H20 or F1-H40. (E) Heatmap representing hierarchical clustering analysis of gene expression profiles between F1-H0 and F1-H20 or F1-H40. Figure S3. Dot plots showing the top 20 most significant Gene Ontology (GO) terms for biological process, cellular component, and molecular function categories in (A) F1-H0 vs. F1-H20 or (B) F1-H0 vs. F1-H40 pairs. GO terms were selected based on an adjusted p-value < 0.05 and a term size between 10 and 500. Figure S4. (A) STRING network diagram showing predicted interactions of CYP26B1. The result shows interactions among CYP family proteins involved in the heme detoxification (summarized in (B)), with CYP1A1 which was also upregulated in the heme-adapted BRMC cells. Figure S5. Comparison of cell viability following shRNA-mediated knockdown of heme-binding genes in control (F1H0, in (A)) or heme-adapted BRMC cells (F1-H20, in (B)) after exposure to increasing concentrations of heme extract for 72 h. Table S1. List of primers used in this study. Table S2. List of top enriched pathways in 20 μM heme adapted BRMC-F2401 cells under DMEM supplemented with 1% FBS.
