# Supplementary material for: Development of a Cost-Effective, Heme-Tolerant Bovine Muscle Cell for Cultivated Meat Production
Source: Foods. 2025 Dec 17;14(24):4348. doi: 10.3390/foods14244348 (PMC12732882; doi:10.3390/foods14244348)
Supplement: Supplementary file 1 [file foods-14-04348-s001.zip › foods-4020366-supplementary.pdf]

Supplementary information

Supplementary Figure S1

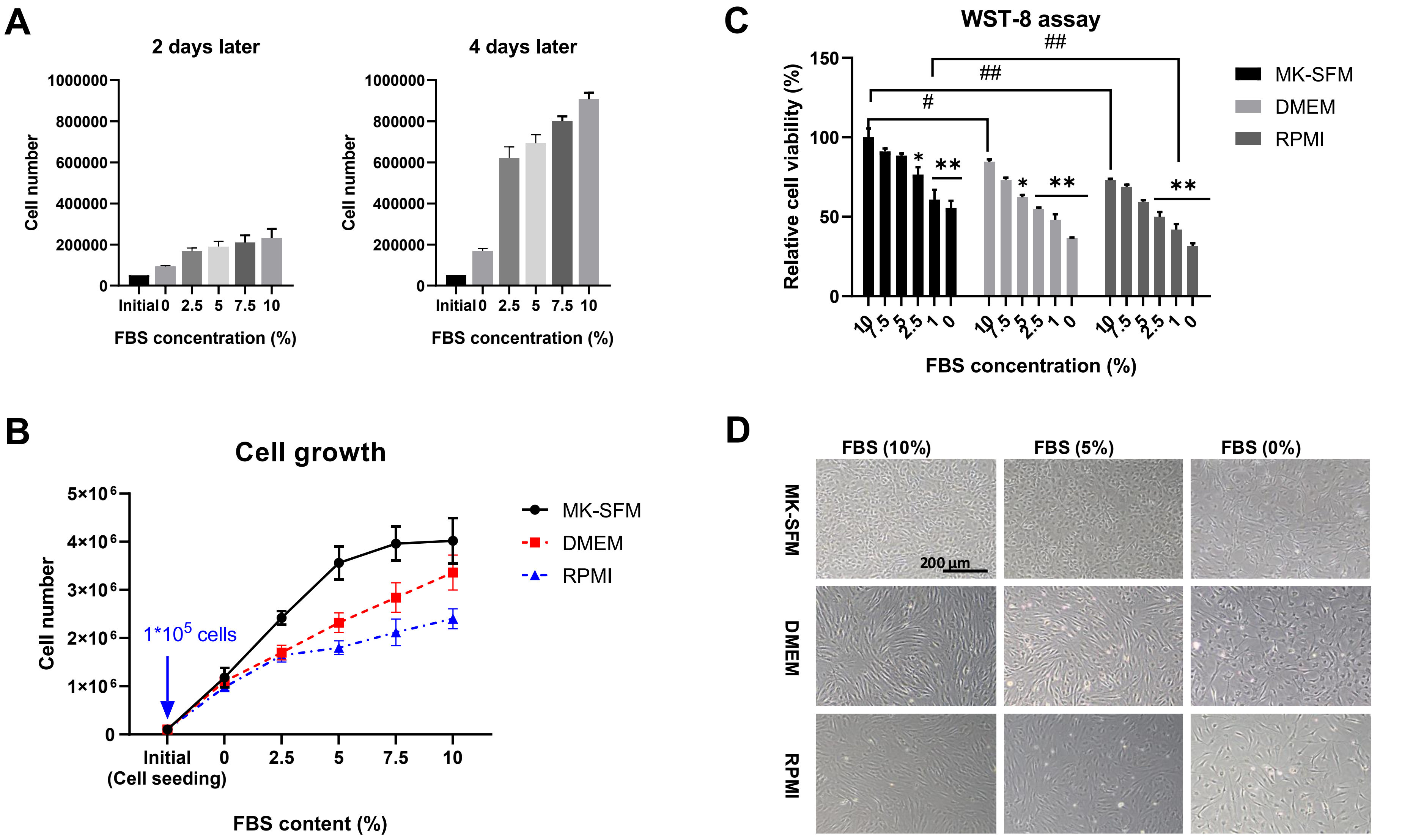

Figure S1. (A) The growth of BRMC in MK-SFM media for 2 and 4 days under various serum conditions. (B) Comparison of BRMC bovine muscle cell proliferation under different culture medium supplemented with increasing amount of fetal bovine serum (FBS). (C) The proliferation of BRMC in three different growth media with decreasing FBS concentration. (D) Morphological changes of cultured BRMC-F2401 cells in three different growth media with serum-free, 5% and 10% FBS concentration.

Supplementary Figure S2

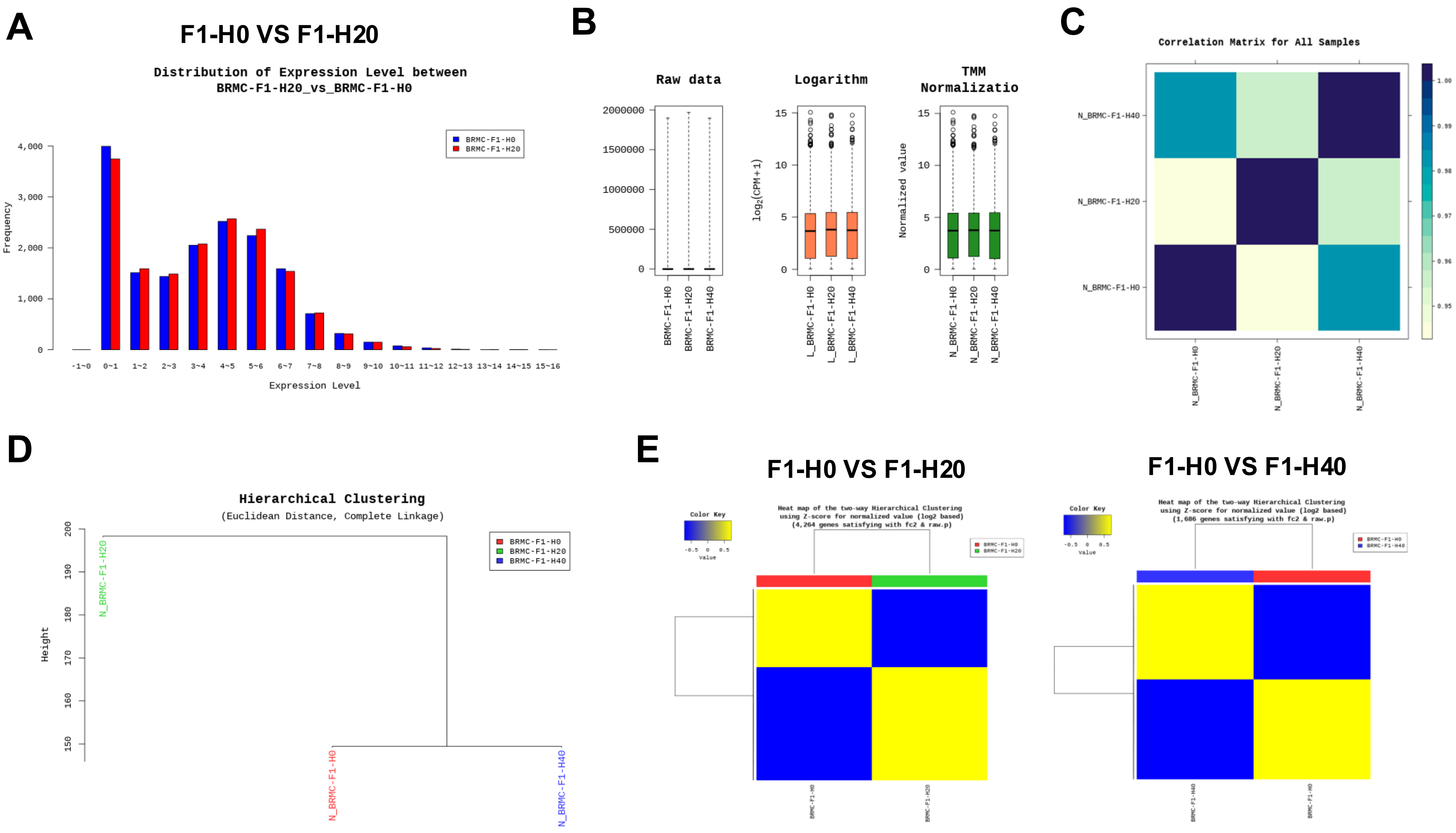

Figure S2. Quality control and analysis of RNA-Seq data from control and heme-adapted BRMC-F2401 cells. (A) Distribution of TMM-normalized values for the pair F1-H0 vs. F1-H20. (B) Boxplots showing expression distributions across samples based on percentiles of raw counts, log_2-transformed read counts, and TMM-normalized values in F1-H0, F1-H20, and F1-H40. (C) Correlation matrix illustrating expression similarity among all samples (F1-H0, F1-H20, and F1-H40). (D) Dendrogram generated from hierarchical clustering of QC-filtered expression data, showing sample similarity between F1-H0 and F1-H20 or F1-H40. (E) Heatmap representing hierarchical clustering analysis of gene expression profiles between F1-H0 and F1-H20 or F1-H40.

Supplementary Figure S3

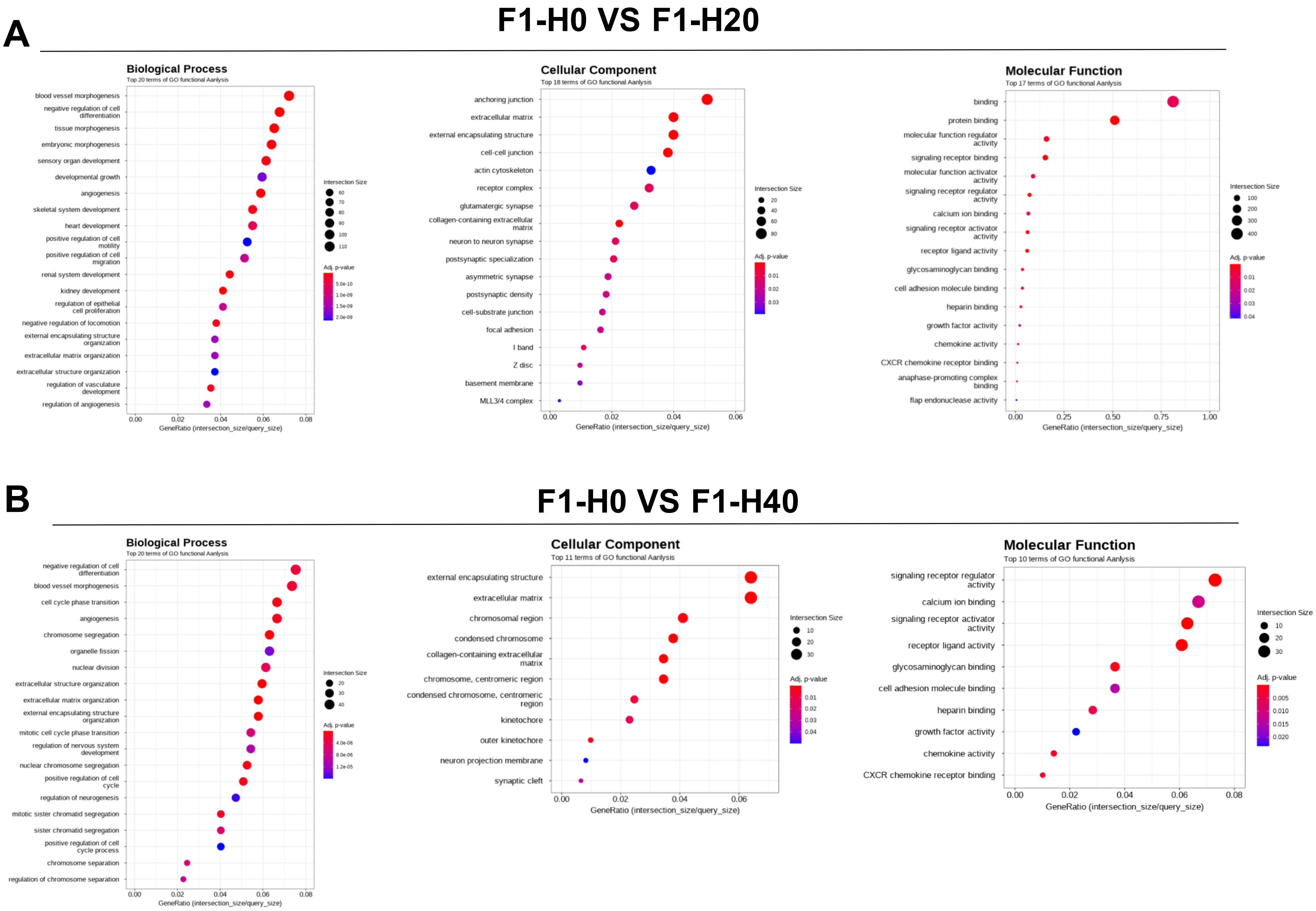

Figure S3. Dot plots showing the top 20 most significant Gene Ontology (GO) terms for biological process, cellular component, and molecular function categories in (A) F1-H0 vs. F1-H20 or (B) F1-H0 vs. F1-H40 pairs. GO terms were selected based on an adjusted *p*-value < 0.05 and a term size between 10 and 500.

Supplementary Figure S4

A

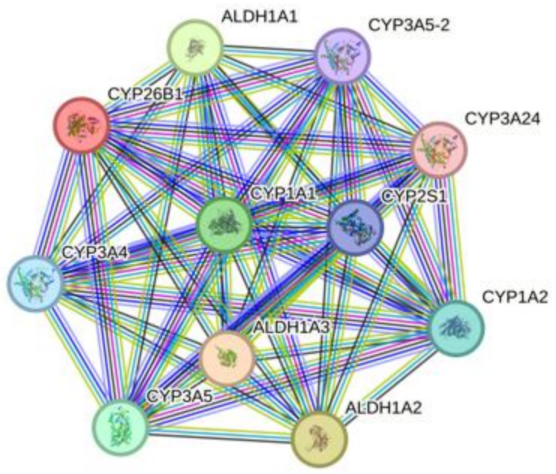

B

Your Input:

CYP26B1	*Cytochrome P450 26B1; Involved in the metabolism of retinoic acid (RA), rendering this classical morphogen inactive through oxidation. Involved in the specific inactivation of all-trans-retinoic acid (all-trans-RA), with a preference for the following substrates: all-trans-RA > 9-cis-RA > 13- cis-RA. Generates several hydroxylated forms of RA, including 4-OH-RA, 4-oxo-RA, and 18-OH-RA. Essential for postnatal survival. Plays a central role in germ cell development: acts by degrading RA in the developing testis, preventing STRA8 expression, thereby leading to delay of meiosis. Required [...] (512 aa)*

Predicted Functional Partners:

		Neighborhood	Gene Fusion	Cooccurrence	Coexpression	Experiments	Databases	Textmining	[Homology]	Score
ALDH1A3	*Aldehyde dehydrogenase 1 family member A3; Belongs to the aldehyde dehydrogenase family.*				●		●	●		0.963
ALDH1A2	*Aldehyde dehydrogenase 1 family member A2; Belongs to the aldehyde dehydrogenase family.*				●		●	●		0.961
ALDH1A1	*Retinal dehydrogenase 1; Can convert/oxidize retinaldehyde to retinoic acid. Binds free retinal and cellular retinol-binding pro...*				●		●	●		0.959
CYP1A1	*Cytochrome P450 family 1 subfamily A member 1; Belongs to the cytochrome P450 family.*			●	●	●	●	●	●	0.940
CYP3A5	*Cytochrome P450 3A28; Cytochromes P450 are a group of heme-thiolate monooxygenases. In liver microsomes, this enzym...*			●	●	●	●	●	●	0.939
CYP1A2	*Cytochrome P450 family 1 subfamily A member 2; Belongs to the cytochrome P450 family.*			●	●	●	●	●	●	0.938
CYP3A4	*Uncharacterized protein.*			●	●	●	●	●	●	0.938
CYP2S1	*CYP2S1 protein; Belongs to the cytochrome P450 family.*			●	●	●	●	●	●	0.934
CYP3A5-2	*Cytochrome P450, family 3, subfamily A, polypeptide 5.*			●	●	●	●	●	●	0.932
CYP3A24	*Uncharacterized protein.*			●	●	●	●	●	●	0.932

Your Current Organism:

Bos taurus

NCBI taxonomy Id: 9913

Other names: B. taurus, Bos bovis, Bos primigenius taurus, Bovidae sp. Adi Nefas, bovine, cattle, cow, dairy cow, domestic cattle, domestic cow

Figure S4. (A) STRING network diagram showing predicted interactions of CYP26B1. The result shows interactions among CYP family proteins involved in the heme detoxification (summarized in (B)), with CYP1A1 which was also upregulated in the heme-adapted BRMC cells.

Supplementary Figure S5

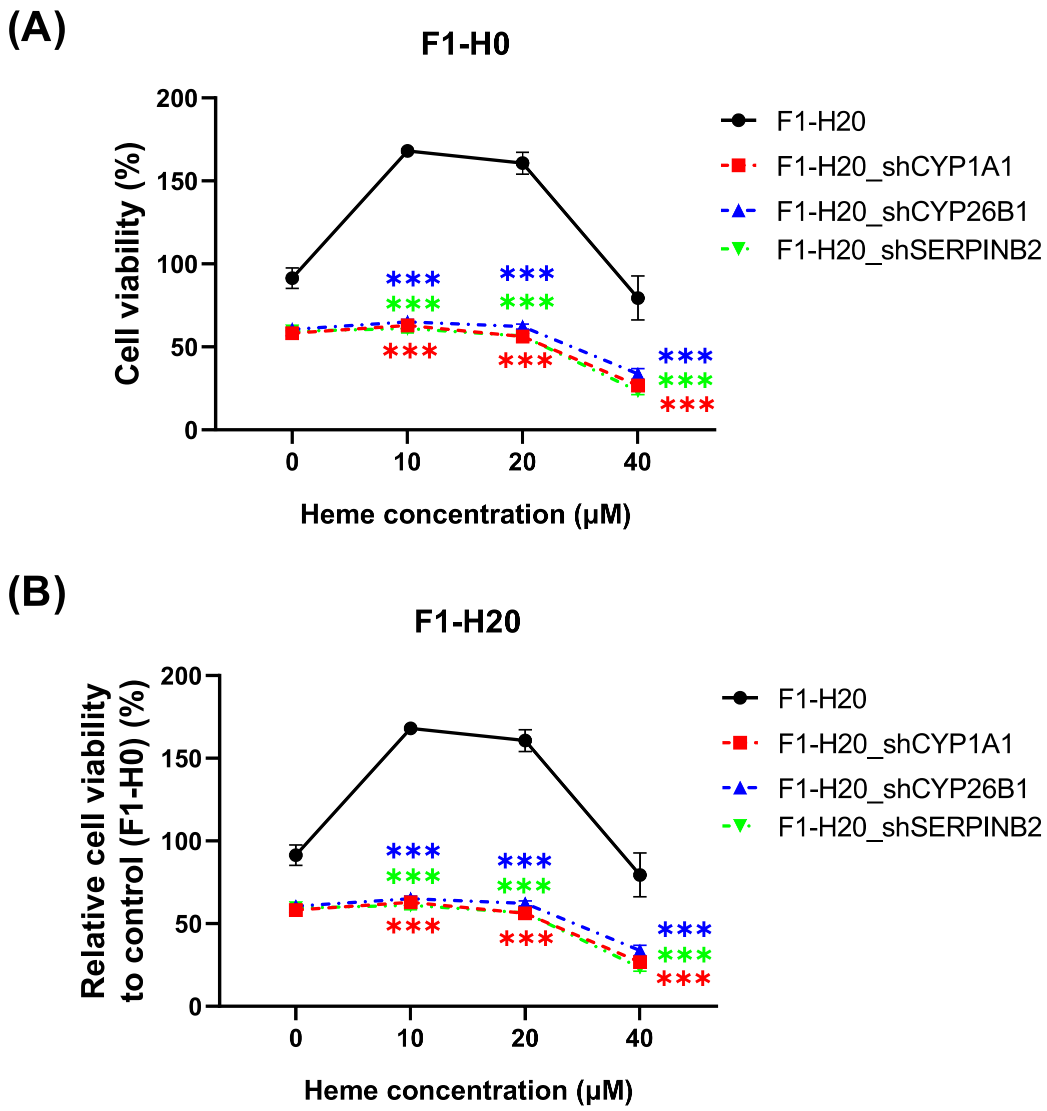

Figure. S5. Comparison of cell viability following shRNA-mediated knockdown of heme-binding genes in control (F1H0, in (A)) or heme-adapted BRMC cells (F1-H20, in (B)) after exposure to increasing concentrations of heme extract for 72 h.

Table S1. List of primers used in this study

Primers for shRNA cloning

Target Gene	Sense	Sequence (5' to 3')
shCYP1A1	Forward	CCGGGCTGGACGAGAATGCCAATATCTCGAGATATTGGCATTCTCGTCCAGCTTTTTG
	Reverse	AATTCAAAAAGCTGGACGAGAATGCCAATATCTCGAGATATTGGCATTCTCGTCCAGC
shCYP26B1	Forward	CCGGGTCCAACTCCATCGGCGATATCTCGAGATATCGCCGATGGAGTTGGACTTTTTG
	Reverse	AATTCAAAAAGTCCAACTCCATCGGCGATATCTCGAGATATCGCCGATGGAGTTGGAC
shSERPINB2	Forward	CCGGGCAACTCTCCAAGAGATATTACTCGAGTAATATCTCTTGGAGAGTTGCTTTTTG
	Reverse	AATTCAAAAAGCAACTCTCCAAGAGATATTACTCGAGTAATATCTCTTGGAGAGTTGC

Primers for qPCR

Target Gene	Sense	Sequence (5' to 3')
CYP1A1	Forward	ATCCCTGTCCTCCGTTACCTG
	Reverse	CTGTGATGTCCCGGATGTGA
CYP26B1	Forward	GCACTATCTGGACTGCGTCA
	Reverse	CGTTCACATCCTTGAACACG
SERPINB2	Forward	CAGGTTCAAACTGGAAGAGCG
	Reverse	GGCCTGTTCTCCCTGTTACG
LOC112441463	Forward	TGCTCATGCTTTTCAACCAG
	Reverse	TCCCATCAATGTCATCCTGA
ALDH1A1	Forward	GAACTCGGGGGAAAGAGTCC
	Reverse	AACACTGGCCCTGGTGATAG
GPX3	Forward	GTCCATGACATCCGGTGGAA
	Reverse	TCTTGACAGAGTTGACCGTGG
CYP2J2	Forward	GTGGCCAGTTATGGAAGGAA
	Reverse	AACGGCATTGTTGATTGTCA
MMP3	Forward	TGCTATGTGTGGCGGTGTG
	Reverse	AGCTTTCCTGTCACCTCCAA
GAPDH	Forward	GACTGGTGTCTGCATGGCTC
	Reverse	GAGTGTCGCTGTTGAAGTCG

Table S2. List of top enriched pathways in 20 μM heme adapted BRMC-F2401 cells under DMEM supplemented with 1% FBS.

term_id	term_name	Adjusted p_value	Term size	Query size	Intersection size	Effective domain_size	Intersection size_UP	Intersection size_DOWN
GO:0048518	positive regulation of biological process	5.27368E-30	4716	1581	675	16190	422	253
GO:0048522	positive regulation of cellular process	1.04862E-29	4441	1581	643	16190	404	239
GO:0048856	anatomical structure development	4.29169E-29	4202	1581	614	16190	341	273
GO:0048731	system development	4.30755E-29	2776	1581	450	16190	253	197
GO:0032502	developmental process	2.03278E-28	4513	1581	645	16190	358	287
GO:0007275	multicellular organism development	1.77291E-27	3285	1581	504	16190	283	221
GO:0051239	regulation of multicellular organismal process	2.66519E-25	2073	1581	352	16190	214	138
GO:0009653	anatomical structure morphogenesis	1.44154E-23	1988	1581	336	16190	177	159
GO:0072359	circulatory system development	2.46546E-23	825	1581	180	16190	105	75
GO:0050789	regulation of biological process	3.19548E-22	9400	1581	1107	16190	654	453
GO:0001944	vasculature development	4.48273E-22	561	1581	137	16190	83	54
GO:0050793	regulation of developmental process	5.81986E-22	1752	1581	301	16190	172	129
GO:0048519	negative regulation of biological process	8.41984E-22	4084	1581	572	16190	347	225
GO:0065007	biological regulation	7.7643E-21	9688	1581	1127	16190	662	465
GO:0035295	tube development	7.7643E-21	820	1581	173	16190	100	73
GO:0001568	blood vessel development	7.7643E-21	534	1581	130	16190	76	54
GO:2000026	regulation of multicellular organismal development	1.62704E-20	996	1581	197	16190	113	84
GO:0035239	tube morphogenesis	2.55772E-20	659	1581	148	16190	89	59
GO:0048523	negative regulation of cellular process	7.09377E-20	3923	1581	546	16190	332	214
GO:0048513	animal organ development	7.92138E-20	2146	1581	342	16190	190	152
